# Stressors in sports coaching: a survey of South African sports coaches

**DOI:** 10.3389/fspor.2025.1687305

**Published:** 2026-01-13

**Authors:** Lesego Phetlhe, Heather Morris-Eyton, Alliance Kubayi

**Affiliations:** 1 Department of Human Movement and Therapeutic Sciences, Tshwane University of Technology, Pretoria, South Africa; 2 Department of Sport and Movement Studies, Faculty of Health Science, University of Johannesburg, Johannesburg, South Africa

**Keywords:** athlete, environment, job insecurity, multiple roles, performance

## Abstract

Sports coaching is a highly stressful profession due to the variety of responsibilities and expectations placed on coaches. The purpose of this study was to assess the sources of this stress among sports coaches in South Africa. The sample consisted of 449 sports coaches (age: 34.6 ± 6.21 years; 274 males and 175 females) who volunteered to participate in the study by responding to the Stressors in Sports Coaching Questionnaire. The results showed that the most important environmental stressors encountered by sports coaches were “condition of playing surface” (*M* = 4.00, *SD* = 0.89), “poorly planned travelling arrangements” (*M* = 4.45, *SD* = 0.80), “distraction while training and/or competing” (*M* = 4.46, *SD* = 0.81), “job insecurity” (*M* = 4.49, *SD* = 0.81), and “unsafe competition arena” (*M* = 4.15, *SD* = 0.68). The major performance-related stressors faced by sports coaches were “being blamed for poor results” (*M* = 4.09, *SD* = 0.72) and “high expectation to win” (*M* = 4.23, *SD* = 0.87). Of the task-related stressors, “performing multiple roles (selection, scouting, etc.)” (*M* = 3.93, *SD* = 0.71) and “making an important decision which later I realised was wrong” (*M* = 3.95, *SD* = 0.78) were reported as most important. These results emphasise the need for coaches to develop psychological skills to help manage the many demands of coaching and thus reduce their stress levels.

## Introduction

Sports coaching has grown into a recognisable profession involving multiple responsibilities and roles (1), from decision-maker and educator to manager, mentor, planner, and sports psychologist (2–4). Sports coaches play a crucial role in promoting participation in sport as well as improving athlete and team performance (3). In addition to their core roles, coaches contribute to the physical, psychological, social, and technical development of athletes as people. Coaches work with diverse populations and face increasing demands from administrators, athletes, parents, and fans (3, 5).

Coaches at all levels of performance are expected to recruit athletes and develop training sessions while coping with organisational (e.g., travelling to events) and performance (e.g., competition outcomes) stressors (6, 7). Additionally, their roles include dealing with a variety of stakeholders such as officials and the media (1, 8). Such demands create complexity and stress in the environments where the coach operates (6, 9). Fletcher et al. (10) define stress as “an ongoing process that involves individuals transacting with their environments, making appraisals of the situation they find themselves in, and endeavouring to cope with any issues that may arise” (p. 329). Stress in the workplace has become increasingly prevalent among sports coaches (1), indicating the growing pressure and demands of the coaching profession. Despite the repercussions associated with stress (9, 10), the stress experienced by sports coaches remains under-investigated. This study seeks to contribute to the body of literature on sources of stress to assist coaches to deal with the demanding nature of their work, lower their stress levels and find a competitive advantage in the South African setting.

Several studies have examined stressors faced by sports coaches worldwide (1, 6, 11). In the United States, Robbins et al. (12) classify the stressors affecting coaches as interpersonal, intrapersonal and contextual. The most frequently cited interpersonal stressors were related to administration, athletes, and expectations from sports organisations and fans. Performance outcomes were the most frequently identified intrapersonal stressors, while lack of resources, job security, and scheduling were the major contextual stressors (12). Other stressors identified by Olusoga and colleagues (13) include athletes' concerns about their performance, competition preparation, and pressure to excel and perform optimally. In South Africa, inadequate facilities, low salaries for coaches, lack of equipment, insufficient support, job insecurity, and poor refereeing decisions were among the stressors experienced by soccer coaches (14).

The few published studies within the South African context contain a small sample size and focus on football to the relative exclusion of other sports (6, 7, 14). This suggests that the results of the previously mentioned studies may not be applicable to coaches of other sports. A deeper understanding of the stressors that various sports coaches face might also help identity those who are likely to experience burnout and consequently quit their jobs. The purpose of this study was thus to assess the sources of stress among South African sports coaches. The findings may be used by sports federations and policymakers to help sports coaches develop intervention strategies for improving their coping mechanisms.

## Research methods

### Participants

The sample consisted of 449 sports coaches (age: 34.6 ± 6.21 years; 274 males and 175 females) who volunteered to participate in the study. The majority of sports coaches were Black (55.5%), followed by 137 (30.5%) who were White, 35 (7.8%) who were People of Colour and 28 (6.2%) Indians. Most coaches were drawn from soccer (24%), rugby (22%), netball (19%), cricket (12%), volleyball (5%), and hockey (5%), with other sports accounting for the remaining 13%.

### Stressors in sports coaching questionnaire (SSCQ)

The SSCQ developed by Kubayi et al. (6) was used to assess the level of stress among sports coaches. The 26-item scale consists of the following four subscales: environmental stressors (10 items, e.g., “condition of playing surface”), performance stressors (4 items, e.g., “Being blamed for poor results”), task-related stressors (6 items, e.g., “performing multiple roles”), and athlete stressors (6 items, e.g., “conflict between athletes”). Each item was scored on a five-point Likert-type scale ranging from 1 (strongly disagree) to 5 (strongly agree). The Cronbach's alpha coefficient value for SSCQ was 0.825, demonstrating that the questionnaire was reliable.

### Data collection

Permission to conduct the study was received from the Research Ethics Committee of the University of Johannesburg (REC-1315-2022). Participants were recruited through their respective sports federations as well as individually, at coaching clinics, seminars, workshops, training sessions, and courses. Upon recruitment, they were informed about the purpose of the study through an information leaflet prior to data collection. The participants received questionnaires from the lead investigator, who also provided them with guidance on how to complete them. Those who were unable to complete the questionnaires in person filled them out online using a Google Doc link that was shared on different social media sites. Completing the questionnaires took from 15 to 20 min.

### Statistical analysis

Descriptive statistics, including means, standard deviations, percentages, and frequency counts, were used to summarise the demographic information about the sports coaches. Prior to conducting the inferential statistics, this study used the Kolmogorov–Smirnov test to check for data normality. Thereafter, the independent *t*-test was used to examine significant differences between male and female sports coaches regarding their sources of stress. Furthermore, Cohen's *d* effect size (ES) was calculated to assess the meaningfulness of the difference and interpreted as follows: 0.20 (small), 0.50 (moderate) and 0.80 (large) (15). The significance level was set at 0.05. All statistical analyses were conducted using SPSS version 28.

## Results

Table 1 shows the sources of stress among between male and female sports coaches. The results showed that the most important environmental stressors they encountered were “condition of playing surface” (*M* = 4.00, *SD* = 0.89), “poorly planned travelling arrangements” (*M* = 4.45, *SD* = 0.80), “distraction while training and/or competing” (*M* = 4.46, *SD* = 0.81), “job insecurity” (*M* = 4.49, *SD* = 0.81), and “unsafe competition arena” (*M* = 4.15, *SD* = 0.68). “Being blamed for poor results” (*M* = 4.09, *SD* = 0.72) and “high expectation to win” (*M* = 4.23, *SD* = 0.87) were their major performance-related stressors.

**Table 1:** Sources of stress amongst coaches according to gender.

Variable	Male	Female	All	ES	Sig.
	*M* (SD)	*M* (SD)	*M* (SD)		
Environmental stressors					
Condition of playing surface	3.97 (0.90)	4.0 (0.87)	4.00 (0.89)	0.03	0.89
Poorly planned travel arrangements	4.45 (0.80)	4.47 (0.81)	4.45 (0.80)	0.02	0.80
Long working hours	3.93 (0.70)	3.96 (0.62)	3.94 (0.67)	0.04	0.67
Distraction while training and/or competing	4.45 (0.82)	4.49 (0.79)	4.46 (0.81)	0.05	0.81
Intimidated by opponents	3.39 (1.14)	3.27 (1.16)	3.35 (1.15)	0.10	1.15
Poor hygiene conditions	4.49 (0.78)	4.57 (0.75)	4.52 (0.76)	0.10	0.76
Job insecurity	4.46 (0.81)	4.53 (0.80)	4.49 (0.81)	0.09	0.81
Travelling long distances	3.68 (0.91)	3.55 (0.85)	3.63 (0.89)	0.15	0.89
My job interfering with family and/or social life	3.86 (0.75)	3.88 (0.70)	3.87 (0.73)	0.03	0.73
Unsafe competition arena	4.15 (0.68)	4.15 (0.68)	4.15 (0.68)	0.00	0.68
Performance stressors					
Being blamed for poor results	4.04 (0.75)	4.15 (0.65)	4.09 (0.72)	0.16	0.72
Financial incentives dependent on results	3.75 (0.88)	3.80 (0.88)	3.77 (0.88)	0.06	0.88
High expectation to win	4.20 (0.89)	4.26 (0.85)	4.23 (0.87)	0.07	0.87
My performance is judged on athletes results	3.83 (0.83)	3.71 (0.74)	3.78 (0.79)	0.15	0.79
Task-related stressors					
Performing multiple roles (selection, scouting, etc.)	3.91 (0.72)	3.95 (0.69)	3.93 (0.71)	0.05	0.71
Managing too many squads	3.38 (0.89)	3.33 (0.83)	3.36 (0.87)	0.06	0.87
Managing other coaches in the programme	3.38 (0.80)	3.30 (0.91)	3.35 (0.85)	0.09	0.85
Lack of recognition for good coaching	3.62 (0.87)	3.61 (0.88)	3.62 (0.88)	0.01	0.88
Making an important decision which later I realized was wrong	3.93 (0.79)	3.99 (0.77)	3.95 (0.78)	0.09	0.78
Interference from management regarding coaching decisions	3.84 (0.90)	3.91 (0.92)	3.87 (0.90)	0.08	0.90
Athlete stressors					
Conflict between me and my athletes	3.81 (0.90)	3.83 (0.91)	3.82 (0.90)	0.02	0.90
Athletes under-performing in training	3.85 (0.89)	3.90 (0.87)	3.87 (0.88)	0.07	0.88
Conflict between athletes	4.06 (0.90)	4.07 (0.87)	4.06 (0.89)	0.01	0.89
Injury to one of my athletes	3.73 (0.92)	3.66 (0.97)	3.71 (0.94)	0.07	0.94
Lack of discipline and commitment from athletes	3.82 (0.94)	3.78 (0.98)	3.81 (0.95)	0.04	0.95

Of the task-related stressors, “performing multiple roles (selection, scouting, etc.)” (*M* = 3.93, *SD* = 0.71) and “making an important decision which later I realised was wrong” (*M* = 3.95, *SD* = 0.78) were reported as major stressors. Of the athlete stressors, only “conflict between athletes” was identified as of major importance (*M* = 4.06, *SD* = 0.89). The subscales perceived as of major importance were environmental stressors, followed by performance, athlete, and task-related stressors. No significant (*p* > 0.05) differences were found between male and female coaches regarding any source of stress.

## Discussion

The findings indicated that the state of the playing field, poor travel plans, distractions during practice or competition, and unsafe competition venues were the main environmental stresses faced by sports coaches. This result aligns with a finding by Kubayi et al. (4) that within the South African context, numerous coaches lacked the necessary sporting facilities to prepare their teams for competition, and those with access had subpar, poorly kept facilities. Similar results have been seen in the UK, where coaches cited stressors related to the accessibility of training resources and facilities (16). Notably, sports coaches spend a significant amount of time recording practice details and competition results to plan future practices and competitions, which can be a source of stress (7).

Another environmental stressor noted by sports coaches was job insecurity, which is in line with findings by Kubayi et al. (17) that indicated a high coach turnover rate across all competition levels. Research has shown that coaches are frequently dismissed by teams that are impatient for success (7). Since sports coaches’ employment contracts are short and their positions precarious (9), it is essential to safeguard their contracts to prevent abrupt dismissal by their clubs or sports organisations without fair compensation. This could help assure sports coaches that their employment is secure through a guarantee that if they are fired before their contracts expire, they will be compensated for the remaining time of their contracts (17).

Sports coaches also reported significant performance-related pressures, including high expectations of success as well as being held accountable for subpar performance. Coaches are generally under pressure to achieve their own performance goals, strive for peak performance, and undergo performance reviews. Like athletes, sports coaches often hold high expectations of themselves, contributing to stress (7, 16). This may be because in South Africa, coaches are often dismissed due to poor performance records or conflicts with team management (18). Notably, high expectations for success may stem from oneself or significant individuals; in South Africa, such pressure often comes from sources such as the media, club owners, and supporters (18).

The primary task-related pressure came from the demand to perform several roles (such as scouting, selection, etc.). Coaches also perceived the time required to plan and conduct practice sessions as a stressor (19). Organising practice sessions, failing to receive credit for effective coaching, planning for competitions, dealing with the consequences of poor decisions, and ensuring every athlete gets enough attention can be overwhelming. Likewise, simultaneously managing several squads or teams often results in task-related stressors (6). Additionally, the pressure to meet deadlines for several teams can increase stress, resulting in a vicious cycle of stress and poor performance (7).

Another significant source of stress for sports coaches was making a decision they subsequently realised was wrong. According to Kubayi et al. (6), the most significant task-related stressors came from being the primary decision-maker and having a great deal of responsibility. This finding may be explained by the fact that coaches must make high-pressure decisions daily (20). Situations that cause uncertainty, such as making substitutions during games (21) or achieving subpar outcomes (20), can impair coaches’ decision-making abilities. As a result, decision-making in coaching is challenging, often requiring consideration of several conflicting factors (21).

## Limitations and future research

The following limitations of the study should be taken into account when interpreting the findings. First, due to the unequal gender distribution of the study sample, the views of female coaches regarding the causes of stress in coaching cannot be taken as representative of all female coaches. Second, the study did not consider demographic variables other than gender when analysing the stressors faced by sports coaches. Future research should use an equal distribution of sports coaches by gender and take into account additional biographical data including age, coaching experience, marital status and coaching level. Third, the study employed only descriptive statistics. Finally, other research methods with qualitative designs (e.g., semi-structured or focus group interviews) should be used in future studies to obtain rich data and comprehensive information about the sources of stress among South African sports coaches.

## Conclusion and practical implications

This study has highlighted the sources of stress encountered by South African sports coaches. The major stressors were environmental stressors, followed by performance stressors, athlete stressors, and task-related stressors. This study also provides practical recommendations for practice in the areas of stress for sports coaches. Sports federations should give coaches access to secure, well-kept facilities to guarantee their athletes' best possible participation. Contracts for sports coaches should include clauses which guarantee job security and prevent termination without fair remuneration. Sports federations and teams should help coaches reduce their workload (scouting, selection, etc.) to allow them to concentrate on the team's performance, thus reducing stress. Incorporating sports psychology into coach education programs will help coaches learn coping skills and stress management techniques. An important role might also be played by informal educational events like conferences and seminars that concentrate on stress-management training of sports coaches.

## Data Availability

The raw data supporting the conclusions of this article will be made available by the authors, without undue reservation.
